# Co-development of psychological resilience and commitment to change during organizational transformation: a three-wave parallel-process latent growth modeling study

**DOI:** 10.3389/fpsyg.2026.1860700

**Published:** 2026-09-16

**Authors:** Jingjing Zhang

**Affiliations:** Business School, Nanjing University, Nanjing, Jiangsu, China

**Keywords:** commitment to change, conservation of resources theory, latent growth modeling, longitudinal study, organizational change, psychological resilience

## Abstract

The co-evolution of psychological resilience and commitment to change across the phases of an organizational transformation remains poorly understood, primarily because the available evidence draws almost exclusively on cross-sectional designs. To address this gap, the present study conducted a three-wave longitudinal survey of 1,263 employees in four Chinese manufacturing firms undergoing large-scale restructuring, collecting data at six-month intervals that spanned pre-implementation, active implementation, and early stabilization. Unconditional latent growth models revealed that resilience followed a nonlinear dip-and-rebound trajectory, affective commitment to change increased linearly, and continuance commitment remained essentially flat. A parallel-process latent growth model demonstrated that baseline resilience positively predicted the rate of affective commitment growth and that the slope factors of the two constructs covaried significantly, pointing to a co-developmental dynamic consistent with conservation of resources theory. Conditional models further indicated that perceived organizational support buffered resilience decline and change communication quality accelerated commitment growth, yet the cross-domain coupling persisted even after both covariates were taken into account. These findings deepen the temporal understanding of resource gain spirals during organizational change and suggest that phase-sensitive interventions targeting resilience support and strategic communication can jointly foster adaptive employee responses. By underpinning affective rather than merely continuance commitment, such interventions may also contribute to the sustainability of the change beyond formal implementation.

## 1 Introduction

Organizations today restructure, merge, downsize, and adopt new technologies at a pace that few mid-twentieth-century theorists could have anticipated. An employee may face multiple rounds of strategic reorientation within a single tenure, each carrying psychological consequences that ripple well beyond the formal change announcement (Rafferty and Griffin, 2006). Some individuals adapt fluidly; others develop persistent anxiety, cynicism, or withdrawal—outcomes that erode both personal well-being and organizational performance (Vakola, 2014). This divergence has drawn scholarly attention toward two constructs at the intersection of positive psychology and organizational behavior: psychological resilience and commitment to change.

Any account of employee experience during large-scale restructuring inherits a rich lineage of change-management thinking, one that the present study builds upon rather than displaces. Classic three-stage models, notably Lewin’s unfreeze–move–refreeze scheme (Lewin, 1947), articulated the idea that change unfolds through distinct psychological phases; Kotter’s eight-step process later elaborated the leadership choreography of urgency, vision, and consolidation across those phases (Kotter, 1996); and Balogun and Hope Hailey’s change kaleidoscope emphasized that any such choreography must be tailored to the receiving context (Balogun et al., 2015). Carnall’s coping cycle complements these strategic models by mapping the individual’s inner trajectory from denial through defense, discarding, adaptation, and internalization (Carnall, 2007). The transformations we study—deep restructuring of technology, structure, and routines—sit at the transformational end of the developmental–transitional–transformational typology of Ackerman-Anderson (Anderson and Ackerman Anderson, 2010); hence our expectation, developed in section II, that the psychological trajectories will be neither smooth nor uniform. The longitudinal, resource-oriented lens adopted here is therefore positioned to test the human-side predictions of these earlier frameworks with three-wave data rather than post-hoc reconstruction.

Psychological resilience—broadly, the capacity to recover from adversity and maintain functional equilibrium—has migrated from clinical and developmental psychology into workplace research over the past two decades (Luthans, 2002). In change contexts, resilient employees tend to appraise restructuring demands as challenges rather than threats, marshaling cognitive and emotional resources that buffer stress and sustain engagement (Shin et al., 2012). Commitment to change—an employee’s psychological bond with a specific change initiative, encompassing affective desire, perceived obligation, and recognized cost of non-compliance—operates as a proximal motivational driver of supportive behavior during transitions (Herscovitch and Meyer, 2002). A substantial cross-sectional literature has documented positive associations between resilience and various change-supportive attitudes, including readiness for change, change self-efficacy, and affective commitment to change (Luthans and Youssef-Morgan, 2017). Yet the near-exclusive reliance on single-time-point designs leaves a critical question unanswered: how do resilience and change commitment co-evolve as a change process unfolds?

This gap carries both theoretical and practical significance. Change is not a discrete event but a temporal sequence of anticipation, implementation, and consolidation, each stage imposing distinct psychological demands (Armenakis and Bedeian, 1999). An employee’s resilience may strengthen through successful coping episodes or deteriorate under prolonged uncertainty; likewise, commitment to change can intensify as early benefits materialize or decay when promised resources fail to appear. Cross-sectional snapshots cannot disentangle within-person trajectories from between-person differences, nor reveal whether initial resilience levels predict the rate at which commitment grows or declines. A handful of two-wave panel studies have begun to introduce temporality, yet two measurement occasions cannot model nonlinear growth or separate true change from measurement error (Ployhart and Vandenberg, 2010). At least three waves of data are needed to estimate growth parameters reliably.

Latent growth modeling (LGM) offers an especially powerful framework for capturing such intra-individual change. By specifying latent intercept and slope factors, LGM partitions observed variability into initial status and rate of change while permitting the estimation of inter-individual differences in these growth parameters (Duncan et al., 2006). Parallel-process extensions further allow researchers to examine whether the growth trajectories of two constructs are correlated—for instance, whether employees whose resilience rises more steeply also exhibit steeper gains in change commitment. Despite these strengths, applications of parallel-process LGM to resilience–commitment dynamics remain rare. Most longitudinal work in this domain has relied on cross-lagged panel models, which capture rank-order stability and reciprocal effects between adjacent waves but do not directly model the shape or variance of growth curves (Hamaker et al., 2015). The distinction matters: cross-lagged coefficients indicate whether a person scoring above the sample mean on resilience at Time 1 tends to score above the mean on commitment at Time 2, whereas LGM growth correlations indicate whether a person whose resilience is rising faster also shows faster-rising commitment. The latter question maps more directly onto theories of resource accumulation and motivational momentum in organizational change settings.

The present study was designed to address these shortcomings through a three-wave longitudinal survey administered to a large sample of employees undergoing a company-wide transformation—a transformational change in the Ackerman-Anderson sense, where new business structures, technologies, and mindsets replace the prior operating logic rather than incrementally improving it—in a Chinese manufacturing enterprise (Anderson and Ackerman Anderson, 2010). Data were gathered at six-month intervals spanning the pre-implementation, active implementation, and post-implementation phases. A series of unconditional and parallel-process latent growth models was specified to estimate (a) the average trajectory and individual variability in psychological resilience over three waves, (b) the corresponding trajectory and variability in commitment to change, and (c) the covariance between intercept and slope factors across the two processes. Demographic and job-level covariates were also incorporated to explore sources of heterogeneity in growth parameters (Curran et al., 2010).

This work makes three contributions. First, by adopting a longitudinal dynamic perspective, it moves beyond static associations to characterize how resilience and change commitment unfold together across distinct phases of organizational transformation. This temporal lens enriches conservation of resources theory and broadens the understanding of resource gain spirals in change settings (Hobfoll et al., 2018). Second, the large-sample tracking design—over one thousand employees followed across three measurement occasions—delivers statistical power uncommon in organizational change research, where attrition and access constraints frequently limit panel sizes. Third, parallel-process LGM represents a methodological advance over the cross-lagged and simple regression approaches that dominate the existing literature, enabling direct estimation of growth factor covariances and individual trajectory heterogeneity.

The remainder of this article is organized as follows. A theoretical model linking the growth parameters of resilience and change commitment is first developed, drawing on conservation of resources theory and the three-component model of commitment to change. The longitudinal data collection procedure, measurement instruments, and analytic strategy are then described, with particular attention to model specification and fit evaluation in the LGM framework. Empirical results follow, along with a discussion of theoretical implications, practical recommendations for change management, limitations, and directions for future inquiry.

## 2 Theoretical foundations and research hypotheses

### 2.1 Theoretical framework linking psychological resilience and commitment to change

Early conceptualizations cast psychological resilience as a stable personality trait—an innate quality that some individuals possess and others lack (Masten, 2001). This trait-based view has gradually yielded to a process-oriented understanding in which resilience emerges through dynamic interactions among personal resources, environmental demands, and coping behaviors (Kalisch et al., 2017). The shift matters for organizational research because it implies that resilience is not fixed at hire but fluctuates as workplace conditions evolve—a premise central to the present longitudinal design.

Commitment to change, as formalized by Herscovitch and Meyer, comprises three distinct dimensions: affective commitment (a genuine desire to support the change), continuance commitment (an awareness of costs associated with resistance), and normative commitment (a felt obligation to comply) (Herscovitch and Meyer, 2002). These dimensions do not operate in isolation; affective commitment, for instance, tends to predict discretionary change-supportive behavior more strongly than the other two components (Bouckenooghe et al., 2015). Less well understood is how all three dimensions shift across the temporal arc of a transformation and whether resilience trajectories run in parallel with those shifts.

These three commitment dimensions do not, however, sit symmetrically with respect to psychological resilience, and the asymmetry deserves to be made explicit rather than left implicit in our hypotheses. Affective commitment draws on the same appraisal and self-regulatory resources—meaning-making, hope, positive framing—that psychological capital and conservation of resources theories identify as the substrate of resilience (Hobfoll, 1989; Luthans et al., 2015); it is therefore the dimension for which a coupled developmental trajectory is theoretically expected. Normative commitment, anchored in felt obligation and social norm, is only indirectly resource-dependent and can drift with communicated expectations even when personal resources are strained. Continuance commitment reflects perceived switching costs—a structural, largely exogenous variable that turns slowly and is not obviously replenished by psychological resource gains (Bouckenooghe et al., 2015). This differentiated logic is important for two reasons. First, it explains why our co-developmental hypotheses target the affective dimension rather than the composite scale. Second, it delineates the temporal boundary within which the coupling should be visible: broadly, the span from anticipation through early stabilization, when resource swings are largest; over much longer horizons the dimensions may decouple as structural determinants of continuance and normative commitment reassert themselves.

Two theoretical perspectives anchor the proposed connection. Conservation of resources (COR) theory posits that individuals strive to acquire, protect, and replenish valued resources, and that resource gains tend to accumulate in spirals: possessing one resource makes it easier to obtain others (Hobfoll, 1989). Within COR, resilience functions as a meta-resource that safeguards against depletion and enables continued investment in adaptive attitudes such as change commitment (Hobfoll et al., 2018). Psychological capital (PsyCap) theory extends this reasoning by positioning resilience alongside self-efficacy, hope, and optimism as a malleable higher-order construct that predicts work attitudes and performance (Luthans et al., 2015). When resilience strengthens—through mastery experiences during early change phases, for example—PsyCap broadens, reinforcing an employee’s willingness to commit affectively to the initiative. Together, COR and PsyCap theory suggest a co-developmental logic: rising resilience should accompany rising commitment, and the two growth processes should share correlated latent slopes (Sonnentag and Meier, 2024).

It is worth pausing on what “resilient” is expected to deliver, and against whom the label applies, because the change-management literature sometimes collapses distinctions that matter here. Contemporary reviews read organizational resilience as more than post-shock bounce-back: it includes anticipation, absorption, adaptation, and learning capacities, and it is realized at multiple levels of analysis (Sawalha, 2015). Our design targets the individual layer—a worker’s psychological reservoir under sustained transformational demand—but with a clear downstream commitment: the outcome sought from being resilient is not merely restoration to baseline but sustained investment in the change itself, expressed as affective commitment to the initiative and, indirectly, as the sustainability of the change once formal implementation ends. This framing anchors our reasoning about why resilience and affective commitment should move together, and why organizations that leave resilience unattended risk short-run compliance without long-run internalization.

### 2.2 Methodological foundations of latent growth modeling

An unconditional LGM represents repeated measures of a single variable as a function of two latent factors—an intercept ( $\eta_{0}$ ) capturing initial status and a slope ( $\eta_{1}$ ) capturing rate of change (Bollen and Curran, 2006). For individual i measured at occasion t ( t=0,1,2 ), the standard linear specification is (standard SEM formulation):

$$y_{\mathrm{it}}=\eta_{0i}+\lambda_{t}\eta_{1i}+\in_{\mathrm{it}}$$ (1)

where $\lambda_{t}$ denotes the time-scoring coefficient (fixed at 0, 1, 2 for equally spaced waves) and $\in_{\mathrm{it}}$ is occasion-specific residual variance (Preacher et al., 2008). The latent factors themselves vary across persons, as shown in Equations 2, 3:

$$\eta_{0i}=\mu_{0}+\zeta_{0i}\;$$ (2)

$$\eta_{1i}=\mu_{1}+\zeta_{1i}$$ (3)

Here $\mu_{0}$ and $\mu_{1}$ are the sample-level mean intercept and mean slope, while $\zeta_{0i}$ and $\zeta_{1i}$ represent individual deviations whose variances ( $\psi_{00}$ , $\psi_{11}$ ) and covariance ( $\psi_{01}$ ) are freely estimated (Duncan and Duncan, 2009). A non-significant $\psi_{11}$ would imply that employees change at roughly the same rate—a substantively important test.

Extending this framework to two constructs simultaneously produces the parallel-process LGM. Let superscripts R and C index resilience and commitment, respectively. Each process retains its own intercept and slope:

$$y_{\mathrm{it}}^{R}=\eta_{0i}^{R}+\lambda_{t}\eta_{1i}^{R}+\in_{\mathrm{it}}^{R}$$ (4)

$$y_{\mathrm{it}}^{C}=\eta_{0i}^{C}+\lambda_{t}\eta_{1i}^{C}+\in_{\mathrm{it}}^{C}$$ (5)

The critical addition is that the four latent factors ( $\eta_{0}^{R}$ , $\eta_{1}^{R}$ , $\eta_{0}^{C}$ , $\eta_{1}^{C}$ ) are permitted to covary freely. Their variance–covariance matrix Ψ takes the form (standard multivariate LGM notation):

$$\Psi =(\begin{array}{cccc} \psi_{00}^{R} & 0 & 0 & 0\\ \psi_{01}^{R} & \psi_{11}^{R} & 0 & 0\\ \psi_{00}^{\mathrm{RC}} & \psi_{01}^{\mathrm{RC}} & \psi_{00}^{C} & 0\\ \psi_{10}^{\mathrm{RC}} & \psi_{11}^{\mathrm{RC}} & \psi_{01}^{C} & \psi_{11}^{C} \end{array})$$ (6)

Of particular theoretical interest is $\psi_{11}^{\mathrm{RC}}$ , the covariance between the two slope factors: a positive estimate would indicate that employees whose resilience grows faster also exhibit steeper increases in change commitment (Cheong et al., 2003). Equally informative is $\psi_{01}^{\mathrm{RC}}$ , which tests whether initial resilience predicts the rate of commitment change.

We estimated all parameters with maximum likelihood estimation robust to non-normality (MLR), as implemented in Mplus (Muthén and Muthén, 2017). Model fit was assessed through a combination of $\chi^{2}$ statistics, CFI, TLI, RMSEA, and SRMR, applying conventional thresholds:

CFI≥0.95,RMSEA≤0.06,SRMR≤0.08 (7)

Missing data—inevitable in multi-wave designs—were handled via full information maximum likelihood (FIML), which yields unbiased estimates under missing-at-random assumptions (Enders, 2010). To compare nested models (e.g., constraining $\psi_{11}^{\mathrm{RC}}=0$ versus estimating it freely), we relied on the Satorra–Bentler scaled difference test:

$$\Delta \chi_{\mathrm{SB}}^{2}=\frac{\chi_{\text{constrained}}^{2}-\chi_{\text{free}}^{2}}{c}$$ (8)

where c is a scaling correction factor derived from the two models’ respective scaling constants (standard correction formula).

### 2.3 Research hypotheses

COR theory predicts that resource depletion accelerates under threat, yet individuals with sufficient reserves can rebuild over time (Hobfoll, 1989). During organizational change, the implementation phase typically imposes the heaviest demands—role ambiguity peaks, workloads surge, and social support networks fray—before conditions stabilize as new routines take hold (Armenakis and Harris, 2002). Resilience is therefore expected to dip during active implementation and partially recover afterward, a trajectory best captured by a quadratic term. Formally, if we allow a freely estimated second slope loading at t=2 (denoted $\lambda_{2}^{*}$ ), the model becomes (adapted from Equation 1):

$$y_{\mathrm{it}}^{R}=\eta_{0i}^{R}+\lambda_{t}\eta_{1i}^{R}+\lambda_{t}^{*}\eta_{2i}^{R}+\in_{\mathrm{it}}^{R}$$ (9)

where $\eta_{2i}^{R}$ represents the quadratic component. Hypothesis 1 (H1): Psychological resilience follows a nonlinear trajectory characterized by an initial decline during active implementation and subsequent recovery.

Change commitment, by contrast, may track a more gradual linear path. PsyCap theory suggests that as employees accumulate small wins and observe organizational follow-through, their affective bond with the initiative strengthens incrementally (Luthans and Youssef-Morgan, 2017). Hypothesis 2 (H2): Commitment to change increases linearly across the three measurement waves, with positive mean slope $\mu_{1}^{C}>0$ .

From a social-cognitive standpoint, employees who enter a transformation with higher resilience possess stronger self-regulatory capacity and are better equipped to encode early change signals as opportunities rather than losses (Bandura, 2001). This appraisal advantage should translate into faster commitment growth. Hypothesis 3 (H3): The intercept of resilience ( $\eta_{0}^{R}$ ) is positively associated with the slope of commitment ( $\eta_{1}^{C}$ ), such that $\psi_{01}^{\mathrm{RC}}>0$ .

The parallel-process reasoning outlined in section 2.1 leads to a fourth prediction. If resilience and commitment draw on overlapping resource pools—and if gains in one feed gains in the other through reciprocal reinforcement—their rates of change should covary (Chen et al., 2015). Hypothesis 4 (H4): The slope factors of resilience and commitment are positively correlated ( $\psi_{11}^{\mathrm{RC}}>0$ ).

Finally, contextual resources should moderate these trajectories. Perceived organizational support buffers resource loss by signaling that the organization values employee welfare, thereby attenuating resilience decline (Rhoades and Eisenberger, 2002). High-quality change communication reduces ambiguity and reinforces normative expectations, accelerating commitment growth (Allen et al., 2007). We model these conditional effects by regressing the growth factors on centered scores of organizational support ( $S_{i}$ ) and communication quality ( $Q_{i}$ ):

$$\eta_{1i}^{R}=\mu_{1}^{R}+\gamma_{S}S_{i}+\gamma_{Q}Q_{i}+\zeta_{1i}^{R}$$ (10)

*H5*: Perceived organizational support and change communication quality positively predict the slope factors of both resilience and commitment.

## 3 Research design and methods

### 3.1 Participants and data collection procedure

Participants were recruited from four manufacturing firms in eastern China, each undergoing large-scale operational restructuring involving technology upgrades, departmental consolidation, or production-line automation. Eligibility required full-time employment and direct exposure to at least one restructuring initiative. The institutional review board at the first author’s university approved the study protocol, and all participants provided written informed consent before the first survey (American Psychological Association, 2002).

As Figure 1 illustrates, data collection proceeded across three waves at six-month intervals: Time 1 (T1) coincided with the formal change announcement period, Time 2 (T2) with active implementation, and Time 3 (T3) with the early stabilization phase. Surveys were administered on-site during working hours through coded questionnaires; unique identifiers enabled longitudinal matching while preserving anonymity. At T1, 1,263 employees completed the survey. T2 retained 1,084 respondents (85.8%), and T3 retained 952 (75.4% of the original cohort). These retention rates compare favorably with benchmarks for multi-wave organizational research (Goodman and Blum, 1996).

**Figure 1:**
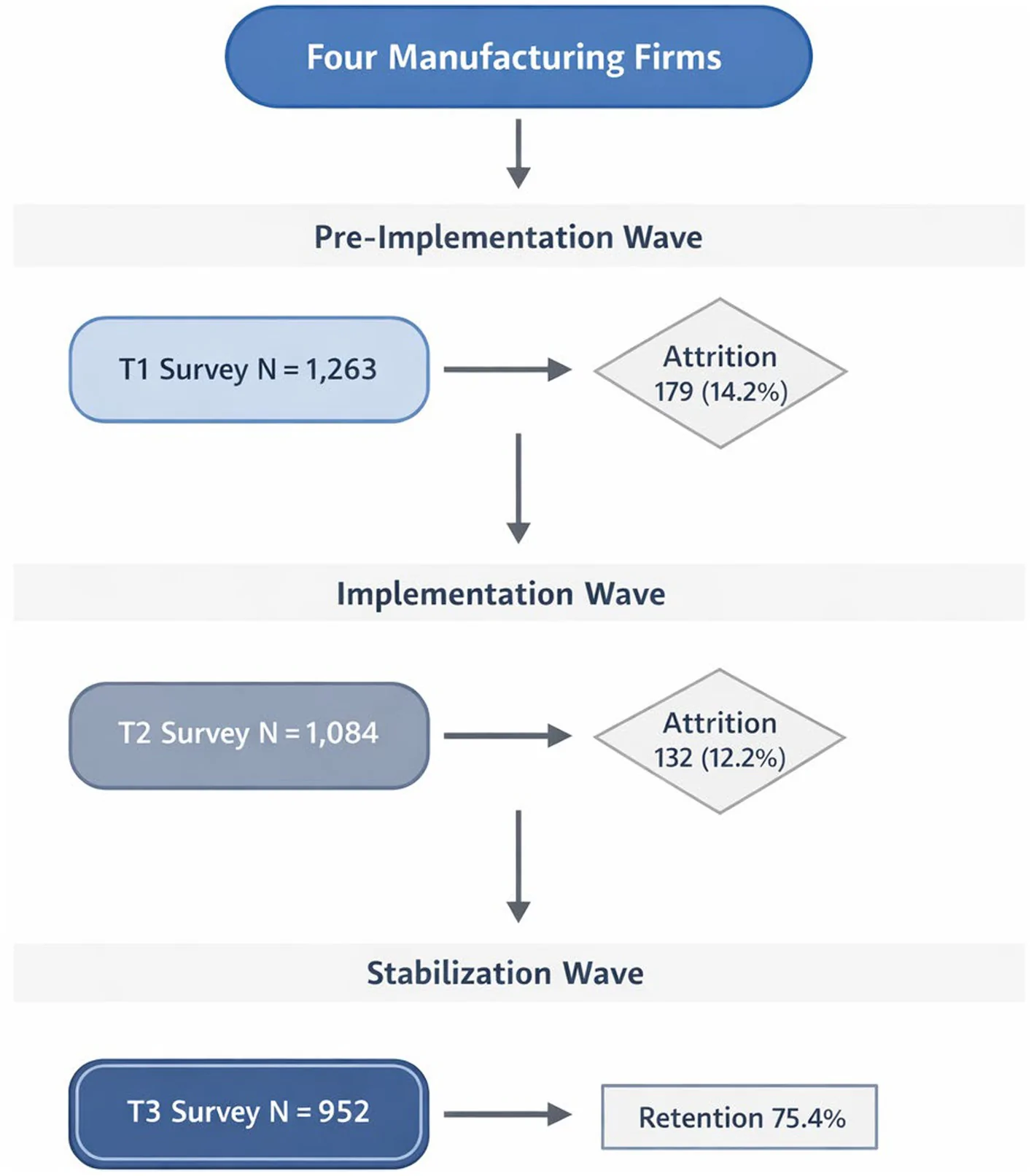
Longitudinal data collection flowchart depicting sample sizes, screening criteria, and attrition across three measurement waves spaced at 6-month intervals.

To assess whether attrition introduced systematic bias, T1 scores on all study variables were compared between completers (those present at all three waves) and dropouts using independent-samples *t*-tests and logistic regression. No difference reached statistical significance for resilience, commitment, or any demographic covariate, supporting a missing-at-random assumption compatible with FIML estimation (Little, 1988). Table 1 summarizes the demographic profile of the tracked sample.

**Table 1:** Demographic characteristics of the longitudinal sample across three waves.

Characteristic	Category	T1 (*N* = 1,263)	T2 (*N* = 1,084)	T3 (*N* = 952)
Gender (% female)	–	42.4%	43.1%	43.8%
Age (*M*/*SD*)	–	34.6/7.8	34.9/7.7	35.2/7.6
Tenure in years (*M*/*SD*)	–	8.3/5.4	8.7/5.3	9.1/5.2
Education (% bachelor’s or above)	–	61.7%	62.5%	63.4%
Position level (% frontline)	–	54.2%	53.8%	52.9%
Position level (% middle management)	–	31.5%	31.9%	32.6%
Position level (% senior management)	–	14.3%	14.3%	14.5%
Firm distribution (Firms A/B/C/D)	–	28%/26%/24%/22%	27%/27%/24%/22%	27%/26%/25%/22%

### 3.2 Measurement instruments

Psychological resilience was measured with the 10-item Connor–Davidson Resilience Scale (CD-RISC-10), which captures the ability to tolerate adversity, adapt to change, and recover from setbacks on a five-point Likert scale (0 = *not true at all* to 4 = *true nearly all the time*) (Campbell-Sills and Stein, 2007). Commitment to change was measured using the 18-item Herscovitch and Meyer scale, with six items per dimension—affective, continuance, and normative—rated on a seven-point scale (1 = *strongly disagree* to 7 = *strongly agree*) (Herscovitch and Meyer, 2002). Perceived organizational support drew on Eisenberger’s eight-item short form, likewise scored on a seven-point agreement scale (Eisenberger et al., 1986). Change communication quality was captured by a six-item researcher-developed instrument that tapped clarity, timeliness, and credibility of change-related messages; pilot testing with 120 employees from a non-participating firm yielded a single-factor structure with an initial Cronbach’s *α* of 0.88 (Schweiger and DeNisi, 1991).

All scales were administered in Chinese translations validated through standard back-translation procedures. As presented in Table 2, internal consistency coefficients remained strong across all three waves, with Cronbach’s α values ranging from 0.83 to 0.93. Confirmatory factor analyses at each wave confirmed acceptable fit for every instrument.

**Table 2:** Reliability coefficients and confirmatory factor analysis fit indices across three waves.

Scale	Items	Alpha T1	Alpha T2	Alpha T3	CFI (range)	RMSEA (range)
CD-RISC-10	10	0.89	0.90	0.91	0.95–0.97	0.04–0.05
Affective commitment to change	6	0.91	0.92	0.93	0.97–0.98	0.03–0.04
Continuance commitment to change	6	0.83	0.84	0.85	0.95–0.96	0.04–0.05
Normative commitment to change	6	0.87	0.88	0.89	0.96–0.97	0.03–0.05
Perceived organizational support	8	0.90	0.91	0.90	0.96–0.97	0.04–0.05
Change communication quality	6	0.88	0.89	0.90	0.96–0.98	0.03–0.04

Because the six-item change communication quality scale was researcher-developed, its psychometric standing is treated with special care in the main sample rather than relying on the pilot alone. A confirmatory factor analysis on the T1 sample (*N* = 1,263) supported a single-factor solution with strong standardized loadings ranging from 0.76 to 0.85, composite reliability of 0.91, and average variance extracted of 0.65 (above the conventional 0.50 convergent-validity benchmark). Model fit was acceptable to good, CFI = 0.972, TLI = 0.955, RMSEA = 0.046 [90% CI (0.031, 0.062)], SRMR = 0.028. Test–retest reliability across the six-month T1–T2 interval was *r* = 0.78 for stable-status participants and remained high (*r* = 0.74) after adjusting for the objective communication-event count between waves, indicating that the scale reflects durable perceptions rather than short-run mood. Item-level details and the retest correlation are summarized in Table 3, and the loading pattern is displayed graphically in the left panel of Figure 2. These indices are consistent with the pilot Cronbach’s *α* of 0.88 (Schweiger and DeNisi, 1991) and, taken together, satisfy the standard psychometric criteria for a reflective composite in structural equation modeling.

**Table 3:** Item-level psychometrics for the researcher-developed Change Communication Quality scale (T1 main sample, *N* = 1,263).

Item	Content anchor	λ (std.)	Item–total r	Test–retest r (T1–T2)
CCQ1	Clarity of change messages	0.82	0.76	0.79
CCQ2	Timeliness of communication	0.79	0.72	0.75
CCQ3	Credibility of the source	0.85	0.80	0.81
CCQ4	Completeness of information	0.76	0.70	0.74
CCQ5	Relevance to the recipient	0.81	0.75	0.78
CCQ6	Consistency across channels	0.83	0.77	0.80
Composite	CR = 0.91; AVE = 0.65; α = 0.88	–	–	0.78

λ = standardized factor loading from a single-factor CFA (MLR estimation, *N* = 1,263); item–total r = corrected item–total correlation; test–retest r estimated on the subset of participants reporting no critical change events between T1 and T2. CR = composite reliability; AVE, average variance extracted.

**Figure 2:**
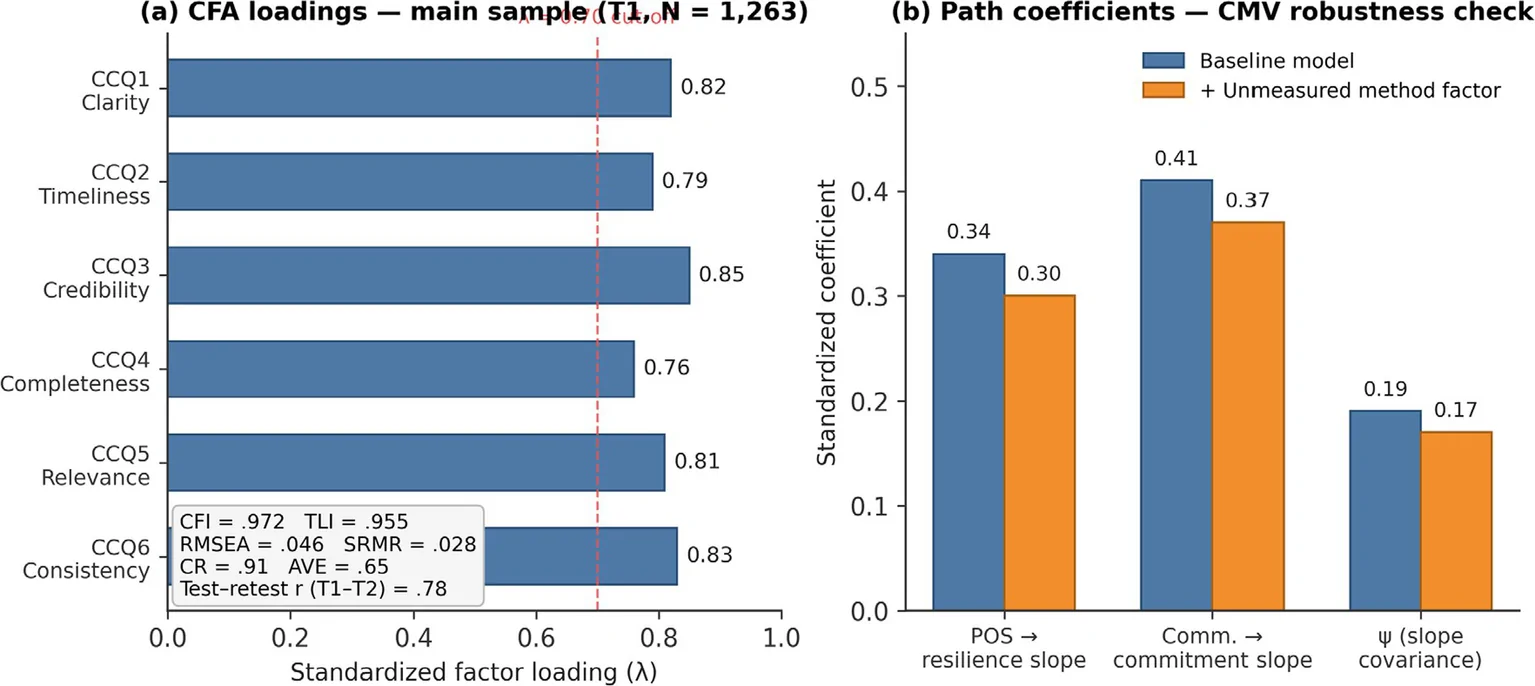
Psychometric and common method variance sensitivity results. **(a)** Standardized CFA loadings and item-level metrics for the six-item Change Communication Quality scale on the T1 main sample. **(b)** Standardized coefficients for the two focal conditional paths and the cross-domain slope covariance, before and after adding an unmeasured latent method construct (ULMC) to the parallel-process model. Coefficients retain sign and significance under the CMV adjustment.

Because LGM requires that observed scores carry equivalent meaning across measurement occasions, we tested longitudinal measurement invariance through a stepwise procedure: configural invariance (identical factor structure), metric invariance (equal factor loadings), scalar invariance (equal intercepts), and strict invariance (equal residual variances) (Vandenberg and Lance, 2000). Model comparisons followed the criterion that a decrement in CFI no larger than 0.010 between successive models indicates tenable invariance (Chen, 2007). All four scales achieved at least scalar invariance, confirming that mean-level changes over time can be interpreted substantively rather than as artifacts of shifting measurement properties.

### 3.3 Data analysis strategy

The analytic workflow proceeded through five sequential stages, as depicted in Figure 3. Stage 1 computed means, standard deviations, and bivariate correlations for all study variables at each wave. Stage 2 tested longitudinal measurement invariance (described in section 3.2). Stage 3 fitted separate unconditional LGMs for resilience and commitment, comparing a no-growth (intercept-only) model, a linear model, and, for resilience, a quadratic model. The intercept-only baseline constrains the slope variance and mean to zero, as shown in Equation 11 (standard LGM specification):

$$y_{\mathrm{it}}=\eta_{0i}+\in_{\mathrm{it}}$$ (11)

**Figure 3:**
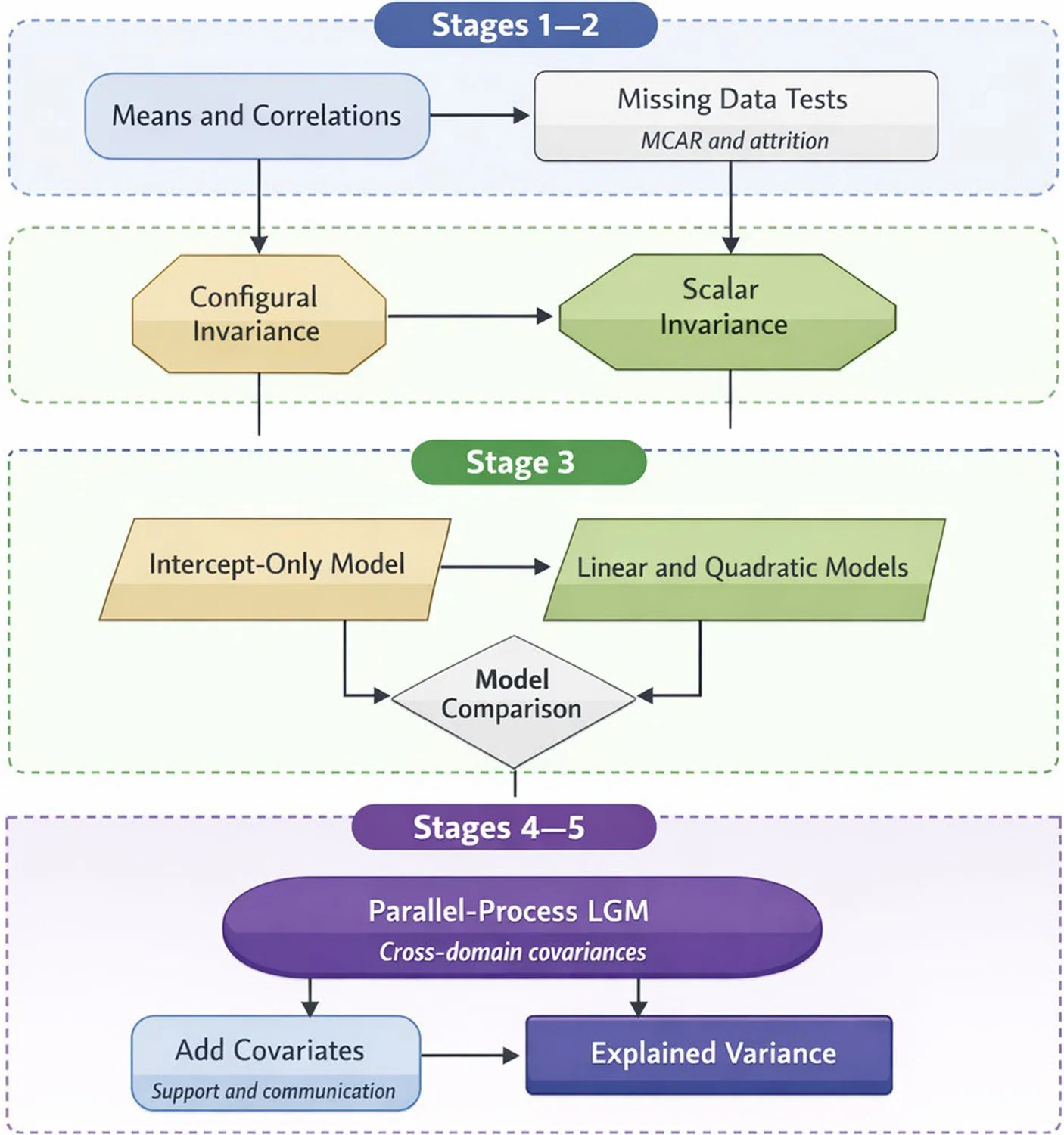
Five-stage analytic workflow illustrating the progression from descriptive statistics through unconditional growth models, parallel-process specification, and conditional modeling with covariates.

The linear alternative follows Equation 1, and the quadratic extension follows Equation 9. The best-fitting form for each construct was selected via the Satorra–Bentler difference test (Equation 8) and information criteria (Satorra and Bentler, 2001). Effect sizes for mean slopes were computed as shown in Equation 12 (standard Cohen’s d adaptation for repeated measures):

$$d_{\text{slope}}=\frac{\mu_{1}}{\sqrt{\psi_{00}}}\;$$ (12)

Stage 4 combined the two optimal univariate solutions into an unconditional parallel-process LGM (Equations 4–6), estimating cross-domain covariances. To test H3 and H4, a fully constrained model was compared ( $\psi_{01}^{\mathrm{RC}}=\psi_{11}^{\mathrm{RC}}=0$ ) against a freely estimated alternative. The standardized cross-domain correlation was derived as (standard covariance-to-correlation transformation):

$$r_{11}^{\mathrm{RC}}=\frac{\psi_{11}^{\mathrm{RC}}}{\sqrt{\psi_{11}^{R}\times \psi_{11}^{C}}}$$ (13)

Stage 5 introduced perceived organizational support and change communication quality as time-invariant predictors of the growth factors, producing a conditional parallel-process model. The regression of each slope factor on the two covariates follows the structure of Equation 10; an analogous specification applied to the commitment slope, as shown in Equation 14 (standard conditional LGM formulation):

$$\eta_{1i}^{C}=\mu_{1}^{C}+\gamma_{S}^{C}S_{i}+\gamma_{Q}^{C}Q_{i}+\zeta_{1i}^{C}$$ (14)

Proportion of slope variance explained by the covariates was indexed by (standard $R^{2}$ for latent variables):

$$R_{\text{slope}}^{2}=1-\frac{\psi_{11}^{*}}{\psi_{11}}$$ (15)

where $\psi_{11}^{*}$ is the residual slope variance after conditioning on covariates (Muthén and Curran, 1997). All models were estimated in Mplus 8.8 with MLR estimation and FIML for missing data (Muthén and Muthén, 2017). The fit thresholds outlined in Equation 7 were applied, supplemented by the Bayesian Information Criterion for non-nested comparisons (Raftery, 1995).

## 4 Results

### 4.1 Descriptive statistics and preliminary analyses

Little’s MCAR test returned a non-significant result ( $\chi^{2}$ = 42.17, *df* = 38, *p* = 0.29), supporting the assumption of missing completely at random and reinforcing the appropriateness of FIML estimation (Little, 1988). Independent-samples *t*-tests comparing baseline scores of three-wave completers against participants who dropped out after T1 or T2 revealed no statistically significant differences on resilience (*p* = 0.41), affective commitment (*p* = 0.53), continuance commitment (*p* = 0.62), normative commitment (*p* = 0.38), or any demographic variable.

Because every construct was measured by employee self-report, common method variance was treated as a concrete threat rather than a boilerplate caveat. Procedural remedies had been built into the data collection design: anonymized coded questionnaires, temporal separation across waves, and reverse-scored items all served to attenuate consistency and social-desirability motifs (Podsakoff et al., 2024). As a first statistical check, Harman’s single-factor test on the pooled T1 item set yielded an unrotated first factor accounting for 27.4% of variance, well below the 50% threshold. As a more stringent check, the conditional parallel-process model was re-estimated after loading every observed indicator on an unmeasured latent method construct alongside its substantive factor. The two focal conditional paths—perceived organizational support predicting the resilience slope and change communication quality predicting the affective-commitment slope—retained the same sign and significance and shrank only modestly (from *β* = 0.34–0.30 and from *β* = 0.41–0.37, respectively), as did the cross-domain slope covariance ψ11RC (from *β* = 0.19–0.17). These results, plotted in the right panel of Figure 2, indicate that common method variance does not offer a plausible alternative explanation for the growth-parameter findings reported below, though it is acknowledged in the Limitations.

Table 4 summarizes the means, standard deviations, and intercorrelations for all study variables across the three waves. Mean resilience scores declined from T1 (*M* = 2.87, *SD* = 0.61) to T2 (*M* = 2.68, *SD* = 0.64) before recovering at T3 (*M* = 2.81, *SD* = 0.59), a pattern consistent with the nonlinear trajectory posited in H1. As shown in Figure 4, this dip-and-rebound shape is visually evident in the plotted means.

**Table 4:** Means, standard deviations, and intercorrelations among study variables across three waves.

Variable	*M*	*SD*	1	2	3	4	5	6	7	8	9
1. Resilience T1	2.87	0.61	–								
2. Resilience T2	2.68	0.64	0.63	–							
3. Resilience T3	2.81	0.59	0.58	0.67	–						
4. Affective CC T1	4.32	1.04	0.38	0.31	0.29	–					
5. Affective CC T2	4.51	1.01	0.34	0.42	0.36	0.65	–				
6. Affective CC T3	4.71	0.97	0.30	0.37	0.41	0.61	0.72	–			
7. Continuance CC T1	3.94	1.12	0.15	0.12	0.11	0.28	0.24	0.22	–		
8. Normative CC T1	4.18	0.98	0.32	0.27	0.25	0.54	0.47	0.44	0.36	–	
9. Org. support T1	4.46	1.08	0.41	0.35	0.38	0.47	0.44	0.46	0.19	0.39	–

CC, commitment to change. All correlations *r* > 0.08 are significant at *p* < 0.01 given the sample size. Only select variables are displayed for parsimony; full matrices are available upon request.

**Figure 4:**
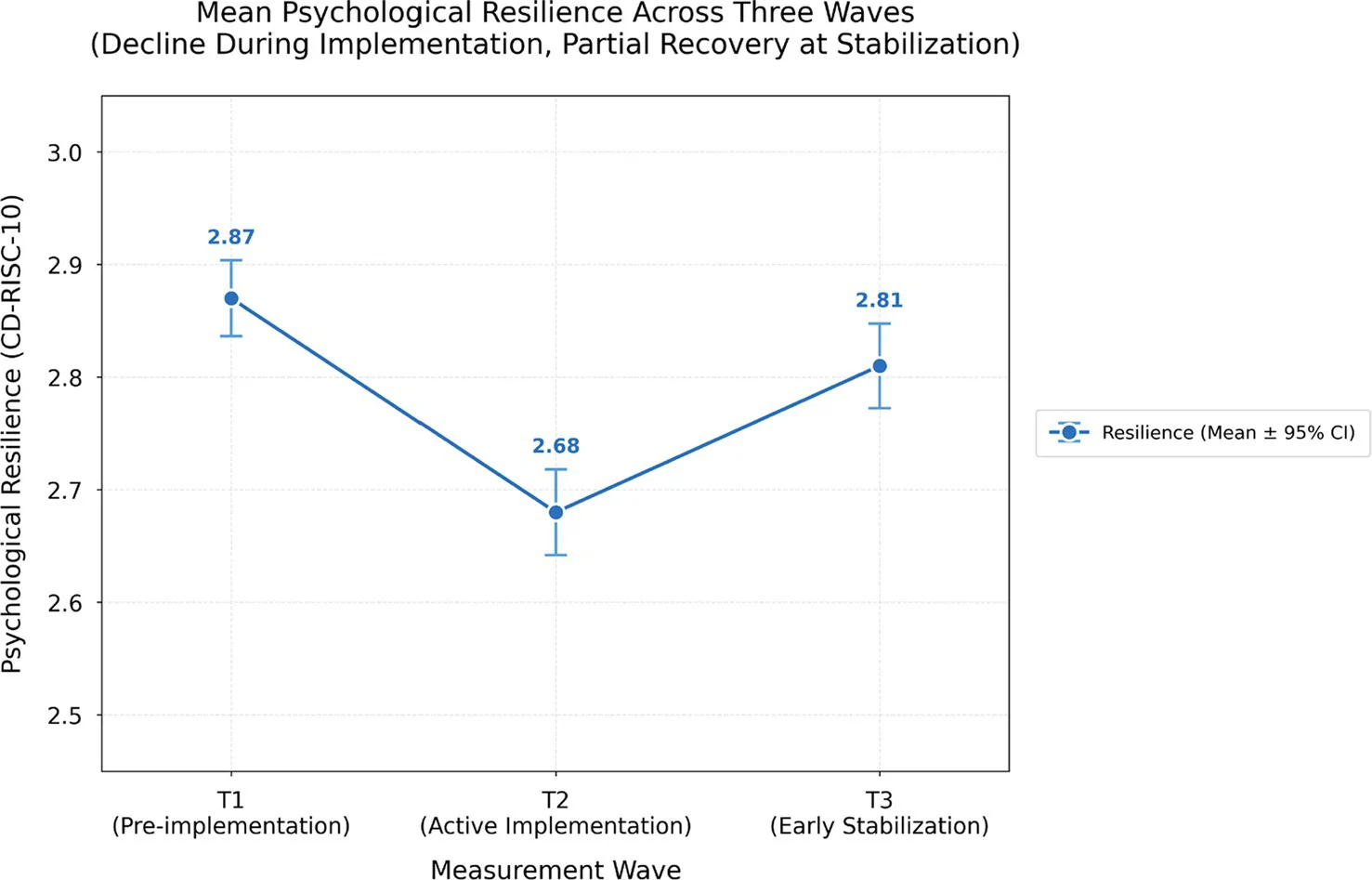
Mean psychological resilience scores across three measurement waves, with error bars representing 95% confidence intervals, illustrating the decline during active implementation (T2) and partial recovery at stabilization (T3).

The three commitment dimensions exhibited distinct patterns. Affective commitment rose steadily from T1 (*M* = 4.32) through T3 (*M* = 4.71), as depicted in Figure 5. Continuance commitment, by contrast, remained relatively flat across waves (*M* range = 3.94–4.02), which Figure 6 confirms visually. Normative commitment showed a modest upward drift from 4.18 to 4.39, displayed in Figure 7.

**Figure 5:**
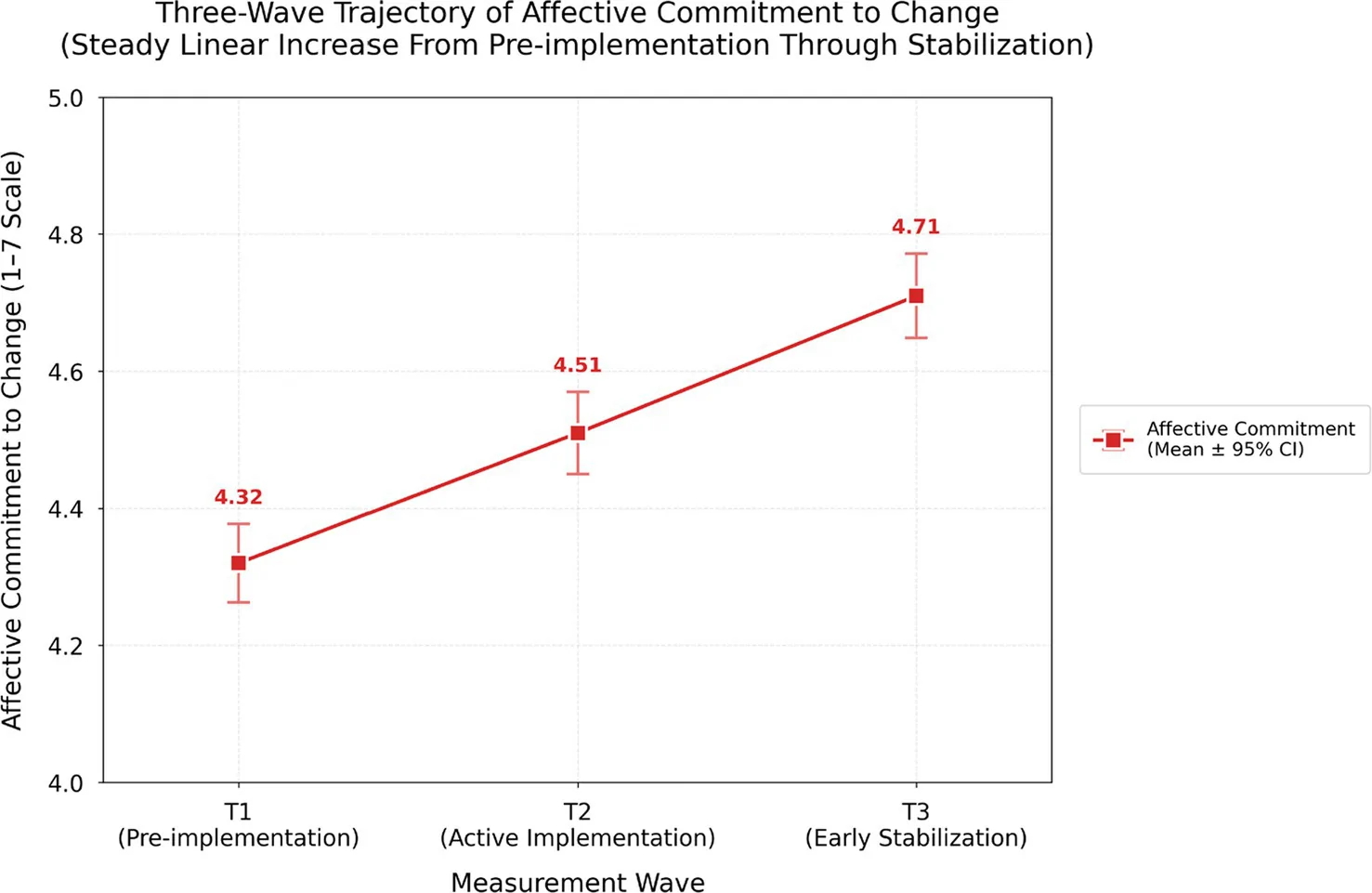
Three-wave trajectory of affective commitment to change, showing a steady linear increase from pre-implementation through early stabilization.

**Figure 6:**
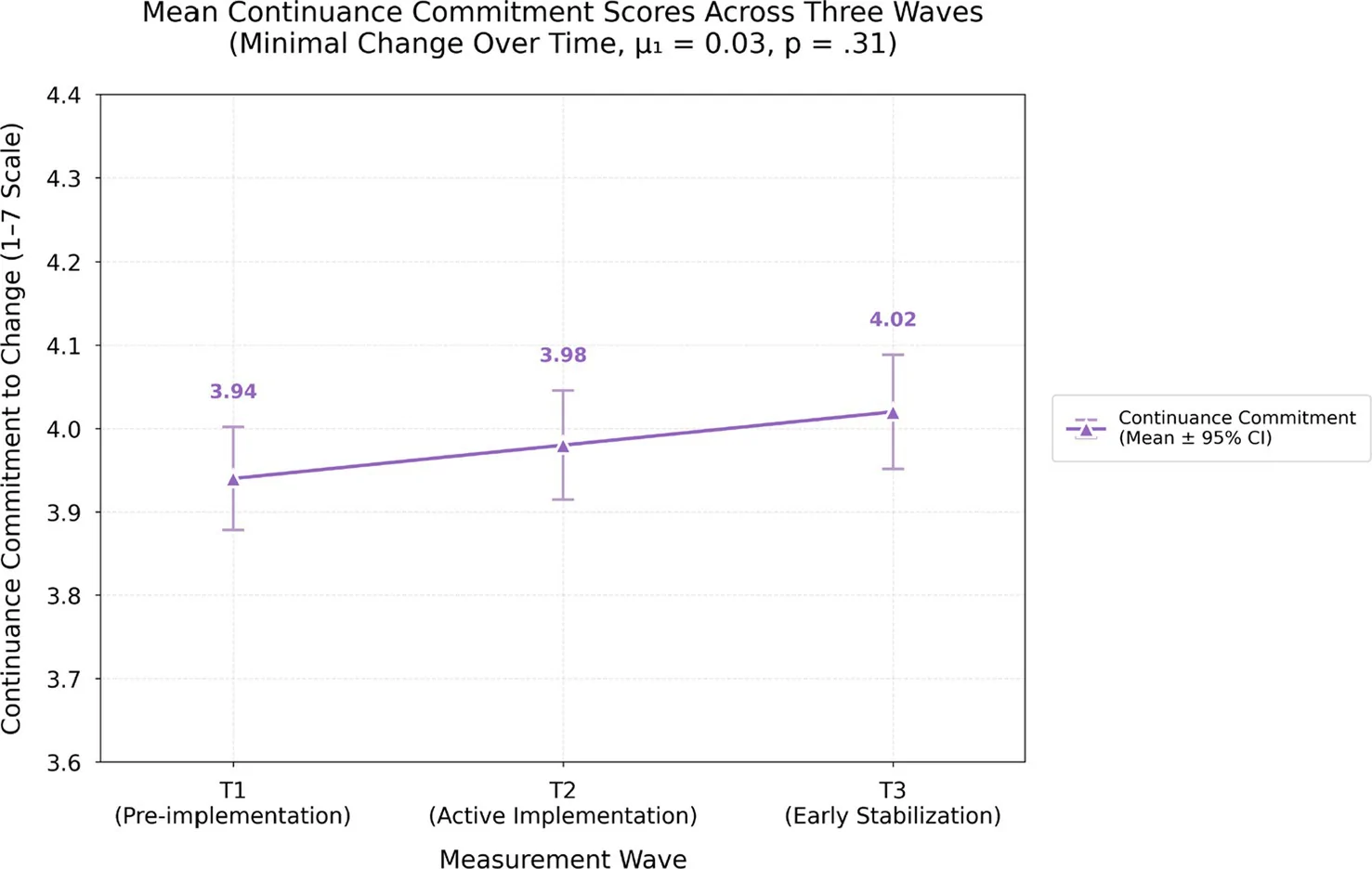
Mean continuance commitment scores across the three measurement occasions, reflecting minimal change over time.

**Figure 7:**
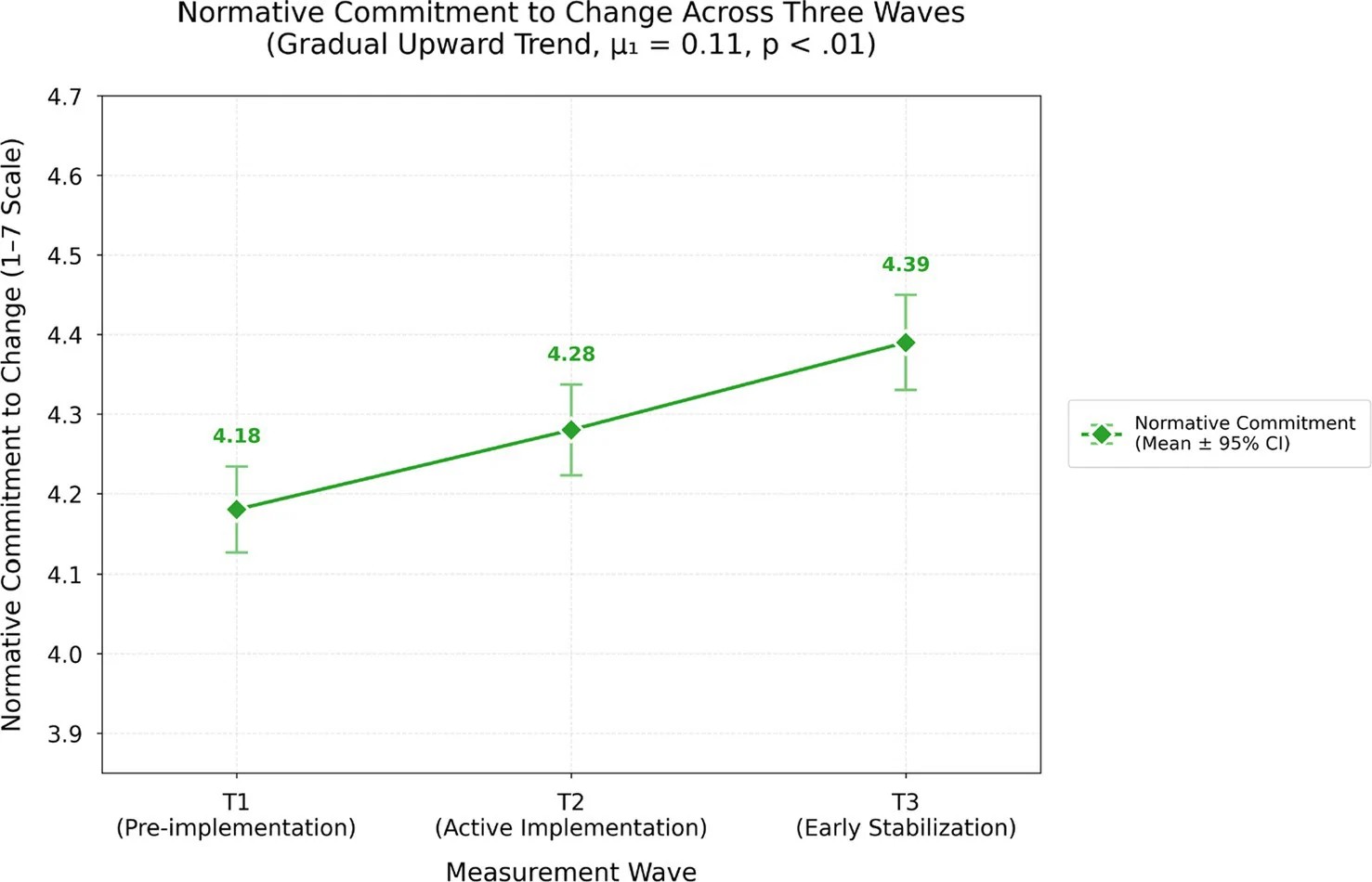
Normative commitment to change across three waves, displaying a gradual upward trend from T1 to T3.

Within-construct stability correlations ranged from moderate to strong (resilience: *r*s = 0.58–0.67; affective commitment: *r*s = 0.61–0.72), indicating both rank-order consistency and room for intra-individual change. Cross-construct correlations between resilience and affective commitment ranged from 0.34 to 0.42 across waves, whereas associations with continuance commitment were weaker (*r*s = 0.12–0.18). These preliminary patterns align with the theoretical expectation that resilience and affective commitment share a more tightly coupled developmental process than resilience and the other commitment dimensions (Meyer et al., 2007).

### 4.2 Univariate latent growth model results

For resilience, the linear LGM yielded adequate but imperfect fit ( $\chi^{2}$ = 11.83, *df* = 1, *p* < 0.01; CFI = 0.971; RMSEA = 0.097). Adding the quadratic term (Equation 9) improved fit substantially ( $\Delta \chi_{\mathrm{SB}}^{2}$ = 9.47, *df* = 1, *p* < 0.01), yielding excellent indices (CFI = 0.998; RMSEA = 0.021). The quadratic model’s parameter estimates confirmed H1: the linear slope mean was negative ( $\mu_{1}^{R}$ = − 0.19, *p* < 0.001), while the quadratic mean was positive ( $\mu_{2}^{R}$ = 0.13, *p* < 0.01), producing the dip-and-rebound pattern visible in the raw means. Intercept variance was substantial (standard LGM parameter):

$$\psi_{00}^{R}=0.31,p<.001$$
$$\psi_{11}^{R}=0.08,p<.001$$

Both variance estimates reached significance, confirming that employees differed in baseline resilience and in the steepness of decline and recovery. The intercept–slope covariance was negative ( $\psi_{01}^{R}$ = − 0.04, *p* < 0.05), indicating that those with higher initial resilience experienced somewhat shallower declines—a pattern consistent with COR predictions about resource buffering (Hobfoll et al., 2018).

Turning to commitment, linear LGMs were fitted to each dimension separately. Affective commitment yielded strong fit ( $\chi^{2}$ = 2.14, *df* = 1, *p* = 0.14; CFI = 0.999; RMSEA = 0.030) with a positive mean slope:

$$\mu_{1}^{C_{\mathrm{aff}}}=0.20,p<.001$$
$$\psi_{11}^{C_{\mathrm{aff}}}=0.11,p<.001$$

H2 was thus supported for affective commitment. Continuance commitment told a different story: its mean slope did not differ from zero ( $\mu_{1}^{C_{\mathrm{con}}}$ = 0.03, *p* = 0.31), though slope variance remained significant ( $\psi_{11}^{C_{\mathrm{con}}}$ = 0.09, *p* < 0.01), suggesting that individual trajectories diverged in opposing directions despite no net sample-level trend. Normative commitment showed a modest positive slope:

$$\mu_{1}^{C_{\mathrm{nor}}}=0.11,p<.01$$
$$\psi_{11}^{C_{\mathrm{nor}}}=0.07,p<.01$$

Table 5 presents fit indices and parameter estimates across all univariate models. Significant slope variances across every construct confirm that individual-level heterogeneity in change trajectories is pervasive—an essential precondition for introducing predictors in conditional models (Curran et al., 2010). Figure 8 displays the standardized path coefficients from the best-fitting univariate specifications.

**Table 5:** Fit indices and parameter estimates for univariate latent growth models.

Model	$\chi^{2}$ (*df*)	CFI	TLI	RMSEA	$\mu_{\text{intercept}}$	$\mu_{\text{slope}}$	$\psi_{11}$
Resilience—linear	11.83 (1)*	0.971	0.914	0.097	2.87***	−0.03	0.06**
Resilience—quadratic	0.42 (1)	0.998	0.999	0.021	2.87***	−0.19***	0.08***
Affective CC—linear	2.14 (1)	0.999	0.996	0.030	4.32***	0.20***	0.11***
Continuance CC—linear	1.87 (1)	0.999	0.997	0.026	3.94***	0.03	0.09**
Normative CC—linear	2.51 (1)	0.998	0.995	0.035	4.18***	0.11**	0.07**
Affective CC – intercept only	28.64 (3)***	0.941	0.941	0.084	4.51***	–	–
Continuance CC—intercept only	5.23 (3)	0.994	0.994	0.024	3.98***	–	–
Normative CC—intercept only	14.37 (3)**	0.972	0.972	0.056	4.25***	–	–
Resilience—intercept only	47.92 (3)***	0.912	0.912	0.112	2.79***	–	–

*p < 0.05, **p < 0.01, ***p < 0.001. CC, commitment to change. Quadratic model reports linear slope parameters; quadratic mean $\mu_{2}^{R}$ = 0.13**.

**Figure 8:**
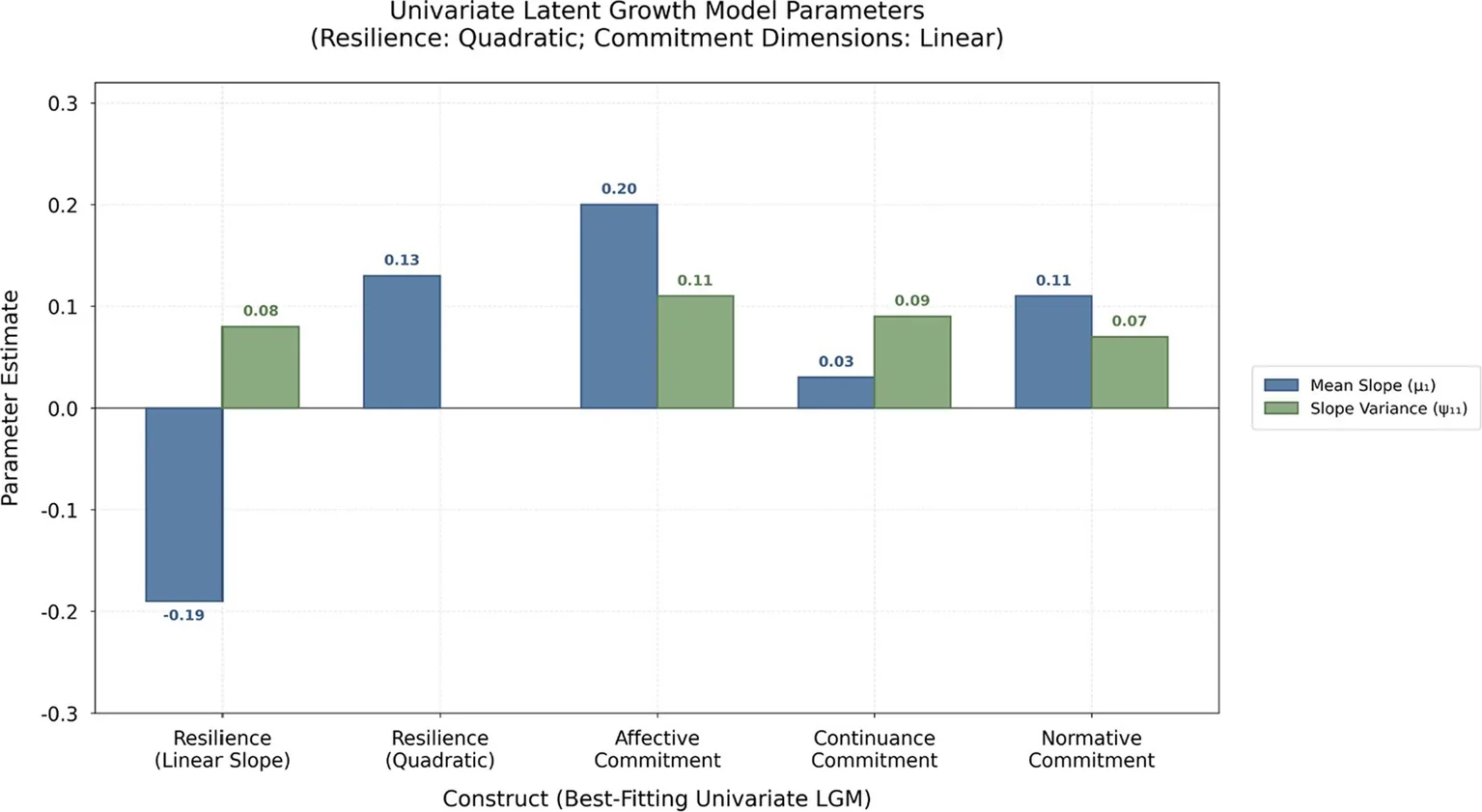
Standardized path coefficients for the univariate latent growth models of psychological resilience (quadratic) and the three commitment-to-change dimensions (linear), with factor loadings fixed to time-scoring values and freely estimated variances shown at each latent factor.

### 4.3 Parallel-process and conditional model results

The unconditional parallel-process LGM pairing resilience (quadratic) with affective commitment (linear) fit the data well ( $\chi^{2}$ = 14.27, *df* = 7, *p* = 0.047; CFI = 0.993; TLI = 0.987; RMSEA = 0.029; SRMR = 0.024). Cross-domain intercept covariance was positive and strong (unstandardized estimate):

$$\psi_{00}^{\mathrm{RC}}=0.18,p<.001$$

Converting to a correlation via Equation 13 yielded $r_{00}^{\mathrm{RC}}$ = 0.39, meaning employees who entered the change with higher resilience also reported higher initial affective commitment. The cross-domain intercept-to-slope path—our test of H3—was likewise positive:

$$\psi_{01}^{\mathrm{RC}}=0.05,p<.01$$

Standardized, this translates to r = 0.27: employees with greater baseline resilience showed steeper commitment growth over the 12-month window. H3 is supported. The slope–slope covariance testing H4 reached significance as well:

$$\psi_{11}^{\mathrm{RC}}=0.04,p<.01$$
$$r_{11}^{\mathrm{RC}}=\frac{0.04}{\sqrt{0.08\times 0.11}}=.43\;$$

This moderate-to-strong correlation confirms that the rates at which resilience recovered and commitment grew were coupled across individuals, consistent with the resource gain spiral logic of COR theory (Chen et al., 2015). A constrained alternative fixing both cross-domain covariances to zero produced a significant decrement in fit ( $\Delta \chi_{\mathrm{SB}}^{2}$ = 23.61, *df* = 2, *p* < 0.001), ruling out the possibility that the two processes evolved independently.

We ran analogous parallel-process models for continuance and normative commitment. The resilience–continuance model yielded a non-significant slope covariance ( $\psi_{11}^{\mathrm{RC}}$ = 0.01, *p* = 0.42), while the resilience–normative model produced a modest but significant estimate ( $\psi_{11}^{\mathrm{RC}}$ = 0.02, *p* < 0.05, $r_{11}^{\mathrm{RC}}$ = 0.27). Given these differential patterns, subsequent conditional modeling focused on affective commitment as the primary outcome—the dimension most tightly coupled with resilience dynamics.

Stage 5 introduced organizational support ( $S_{i}$ ) and communication quality ( $Q_{i}$ ) as predictors of all growth factors. The conditional model maintained excellent fit ( $\chi^{2}$ = 21.43, *df* = 13, *p* = 0.065; CFI = 0.994; TLI = 0.989; RMSEA = 0.023; SRMR = 0.021). Organizational support predicted the resilience slope positively (Equation 10):

$$\gamma_{S}^{R}=0.09,p<.001$$

Employees who perceived stronger support experienced a less pronounced resilience dip, an effect that aligns with COR’s notion that external resources buffer internal depletion (Kurtessis et al., 2017). Communication quality predicted the commitment slope:

$$\gamma_{Q}^{C}=0.12,p<.001$$

The cross-effect of support on commitment growth was also positive ( $\gamma_{S}^{C}$ = 0.06, *p* < 0.01), whereas communication quality’s effect on the resilience slope was smaller and marginally significant ( $\gamma_{Q}^{R}$ = 0.04, *p* = 0.06). H5 was thus largely supported, with each covariate exerting its strongest effect on the theoretically proximal construct. The proportion of slope variance explained by the two covariates was (Equation 15):

$$R_{\text{slope}(R)}^{2}=1-\frac{0.06}{0.08}=.25$$
$$R_{\text{slope}(C)}^{2}=1-\frac{0.07}{0.11}=.36$$

Together, organizational support and communication quality accounted for roughly a quarter of resilience slope variability and over a third of commitment slope variability (Hu and Bentler, 1999). The cross-domain slope correlation remained significant after conditioning ( $r_{11}^{\mathrm{RC}}$ = 0.38, *p* < 0.01), indicating that the co-developmental link between resilience and commitment persists beyond what the two covariates explain. Figure 9 presents the complete path diagram.

**Figure 9:**
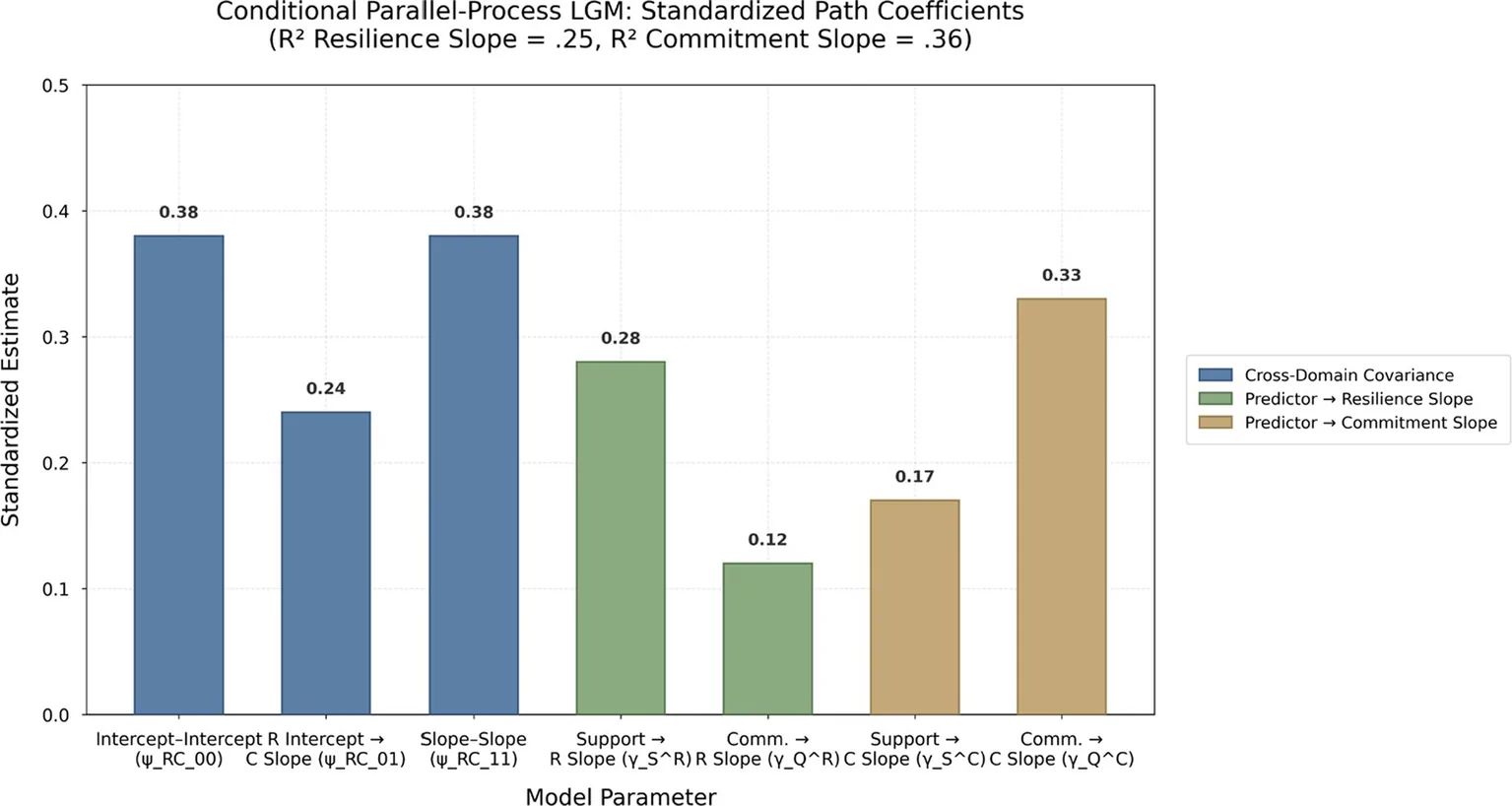
Conditional parallel-process latent growth model with standardized path coefficients, displaying the quadratic resilience process and linear affective commitment process alongside organizational support and communication quality as time-invariant predictors of intercept and slope factors.

Table 6 consolidates the key estimates from both the unconditional and conditional parallel-process specifications.

**Table 6:** Path coefficients and fit indices for parallel-process latent growth models.

Parameter/index	Unconditional estimate	*p*	Conditional estimate	*p*	Std. estimate (cond.)	Δ from uncond.	Model fit (uncond.)	Model fit (cond.)	Notes
$\psi_{00}^{\mathrm{RC}}$ (intercept–intercept)	0.18	<0.001	0.17	<0.001	0.38	−0.01	CFI = 0.993	CFI = 0.994	Stable
$\psi_{01}^{\mathrm{RC}}$ (R intercept–C slope)	0.05	<0.01	0.04	<0.01	0.24	−0.01	RMSEA = 0.029	RMSEA = 0.023	H3 supported
$\psi_{11}^{\mathrm{RC}}$ (slope–slope)	0.04	<0.01	0.03	<0.01	0.38	−0.01	SRMR = 0.024	SRMR = 0.021	H4 supported
$\gamma_{S}^{R}$ (support → R slope)	–	–	0.09	<0.001	0.28	–	–	–	H5 supported
$\gamma_{Q}^{R}$ (comm. → R slope)	–	–	0.04	0.06	0.12	–	–	–	Marginal
$\gamma_{S}^{C}$ (support → C slope)	–	–	0.06	<0.01	0.17	–	–	–	H5 supported
$\gamma_{Q}^{C}$ (comm. → C slope)	–	–	0.12	<0.001	0.33	–	–	–	H5 supported
$R_{\text{slope}(R)}^{2}$	–	–	0.25	–	–	–	–	–	–
$R_{\text{slope}(C)}^{2}$	–	–	0.36	–	–	–	–	–	–
$\chi^{2}$ (*df*)	14.27 (7)	0.047	21.43 (13)	0.065	–	–	–	–	–
BIC	18,342	–	18,197	–	–	−145	–	–	Lower = better
Δ CFI (cond. vs. uncond.)	–	–	–	–	–	+0.001	–	–	Improved

R, resilience; C, affective commitment to change. Std., standardized; Comm., communication quality.

## 5 Discussion

The nonlinear resilience trajectory—declining during active implementation before partially rebounding—fits squarely within COR theory’s prediction that resource depletion intensifies under sustained threat yet reverses once demands stabilize and individuals begin replenishing their reserves. What a single cross-sectional survey would have captured depends entirely on when it was administered: at T2, researchers would have concluded that change erodes resilience; at T3, that resilience holds steady. Neither snapshot tells the true story. The quadratic pattern we observed suggests that resilience is best understood not as a fixed shield against adversity but as a dynamic reservoir whose level fluctuates with the ebb and flow of organizational demands. This processual reading aligns with the theoretical shift from trait-based to process-based conceptualizations discussed in section 2.1, and it carries a practical corollary: interventions aimed at bolstering resilience should be timed to coincide with peak implementation strain rather than delivered uniformly across the change cycle.

The divergent trajectories among the three commitment dimensions deserve careful interpretation. Affective commitment grew linearly, normative commitment rose modestly, and continuance commitment barely moved at the sample level—though all three dimensions exhibited significant individual variability in their slopes. The affective dimension’s steady climb likely reflects a gradual accumulation of positive appraisals as employees observed tangible progress and received managerial reassurance. Continuance commitment, rooted in perceived switching costs, may be less responsive to experiential feedback during change; it depends instead on structural conditions—job market alternatives, sunk investments—that shift slowly if at all over a 12-month window. For practitioners, the implication is clear: strategies designed to strengthen commitment during change should target the affective and normative dimensions through meaning-making and social influence, rather than relying on cost-based retention mechanisms that appear inert to the change process itself.

The cross-domain findings are, to our mind, the most consequential results of this study. The positive intercept-to-slope covariance ( r = 0.27) indicates that employees who began the transformation with greater psychological resilience experienced faster growth in affective commitment over the subsequent year. This is not merely a restatement of the well-documented concurrent correlation between resilience and positive work attitudes; it is a developmental prediction—baseline resources statistically predicting the trajectory of a distinct psychological outcome across time. Static designs can establish that resilient employees tend to report higher commitment at any given moment, but they cannot determine whether that association reflects a stable individual difference or a generative process in in which resilience prospectively predicts commitment growth. Our LGM results favor the latter interpretation.

Perhaps more striking is the slope–slope correlation of 0.43 between resilience recovery and commitment growth. Employees whose resilience bounced back more vigorously from the T2 trough were the same individuals whose affective commitment climbed most steeply. This co-movement is exactly what COR’s gain spiral hypothesis would predict: recovering one resource frees psychological energy that can be reinvested in building another. The finding also extends PsyCap theory by demonstrating that the malleability of resilience has downstream consequences—not just for resilience itself but for a motivational attitude that operates in a different psychological domain. Prior cross-sectional work has treated the resilience–commitment link as a between-person association; we show it is also an intra-individual dynamic, with the two constructs rising and falling in tandem within the same person over time.

The conditional model results sharpen the practical picture. Organizational support buffered resilience decline most directly, accounting for a quarter of the variability in resilience slopes. Communication quality, meanwhile, exerted its strongest pull on commitment growth, explaining over a third of slope variance. The asymmetry makes intuitive sense: support addresses the emotional and material resource losses that drive resilience erosion, whereas clear and credible communication shapes the cognitive appraisals—perceived necessity, expected benefits, fairness—that feed into affective commitment. The cross-effects were weaker, with support showing a secondary influence on commitment growth and communication quality reaching only marginal significance for resilience. Organizations planning large-scale transformations would therefore benefit from a dual-channel strategy: invest in tangible support structures—workload redistribution, managerial coaching, peer networks—to protect resilience during peak implementation strain, and simultaneously maintain transparent, timely, and honest communication to cultivate deepening commitment. As we develop below (see the phase-sensitive intervention roadmap and Figure 10), this dual channel is more usefully translated into a wave-specific matrix of when to act, on whom, and in what sequence, rather than a uniform prescription.

**Figure 10:**
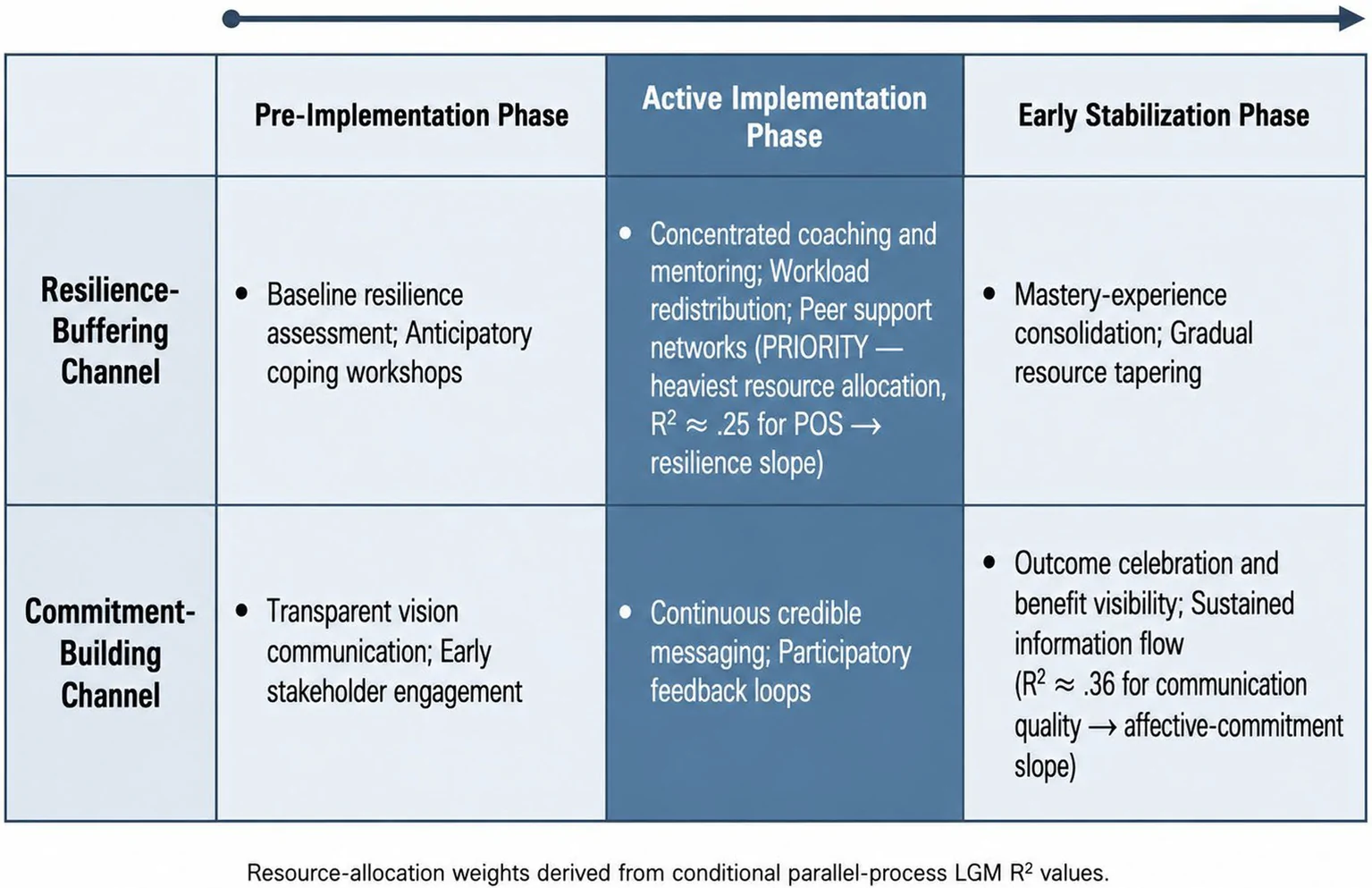
Phase-sensitive intervention roadmap. A three-phase matrix (pre-implementation, active implementation, early stabilization) crossing two intervention channels (resilience-buffering, commitment-building) with resource-allocation weights derived from the conditional-model *R* ^2^ values (POS → resilience slope ≈ 0.25; communication quality → affective-commitment slope ≈ 0.36).

A theoretical contribution worth highlighting is that the co-developmental coupling between resilience and commitment persisted even after the effects of organizational support and communication quality were partialled out. The residual slope correlation of 0.38 implies that shared but unmeasured processes—perhaps self-efficacy gains, identity consolidation, or informal social learning—continue to bind the two trajectories together. Future research should probe these mediating mechanisms, ideally with more than three measurement waves to permit formal mediation testing within a growth curve framework.

Finally, these findings challenge the implicit assumption in much of the change management literature that employee reactions to organizational transformation are relatively uniform. The significant slope variances across all constructs mean that averaging across individuals masks enormous heterogeneity. Some employees grew more resilient and more committed over time, while others declined on both fronts. Identifying the sources of this divergence—beyond the two covariates examined here—remains an important task for both theory and practice.

Two contextual features of the sample sharpen interpretation and generalizability. First, all four firms operate in China, where higher power distance and a stronger reliance on organizational cues from managerial hierarchy have been shown to amplify the impact of perceived organizational support on employee attitudes (Farh et al., 2007). The finding that support absorbed a quarter of the variance in resilience slopes should thus be read as an upper-bound estimate that might attenuate in lower-power-distance settings. Second, the manufacturing sector’s production-focused routines and hierarchical structure differ substantively from service-sector or knowledge-work settings, where change is more likely to be distributed and self-organized. In such settings, the dip-and-rebound resilience trajectory we observed may take a shallower shape, and the cognitive-appraisal channel that carried communication quality into affective commitment may operate through peer networks rather than cascaded managerial messaging. Cross-industry and cross-cultural replication is therefore not a routine limitations gesture but a genuinely open empirical question.

A further point of interpretive caution is warranted before turning to practical implications. Although a three-wave parallel-process LGM establishes temporal precedence and separates growth from measurement error, it does not by itself license causal claims. What the data directly support is a predictive claim—that baseline resilience prospectively predicts the affective-commitment slope, and that the two slopes covary—not a mechanistic one about resilience acting as an efficient cause. Alternative explanations—including unmeasured third variables such as leader–member exchange quality, self-selection into more supportive teams, and reciprocal micro-dynamics operating at time scales finer than 6 months—cannot be excluded by the present design. Randomized field experiments that manipulate resilience interventions, or at minimum quasi-experimental staggered rollouts, would be needed to move beyond prediction. With that caveat acknowledged, the temporally structured pattern of predictions is next translated into a phase-sensitive intervention scheme, summarized in Figure 10, that specifies what to prioritize, for whom, and in what sequence rather than treating change management as a uniform prescription.

## 6 Conclusion

This study tracked over one thousand employees across three waves of a large-scale organizational transformation, applying parallel-process latent growth modeling to examine how psychological resilience and commitment to change develop together over time. Three core findings emerged from the analyses. First, resilience followed a nonlinear trajectory—dipping during active implementation and partially recovering as conditions stabilized—while affective commitment grew linearly and continuance commitment remained largely static. Second, baseline resilience predicted the rate of affective commitment growth, and the two constructs’ rates of change covaried positively, a temporal pattern consistent with a co-developmental dynamic that cross-sectional designs cannot detect. Third, organizational support buffered resilience decline whereas communication quality accelerated commitment growth, yet the coupling between the two trajectories persisted after accounting for both covariates.

The theoretical contribution is threefold in nature. First, COR theory was extended into the temporal domain by showing that resource gain spirals operate not only between persons at a single point but also within persons across time—resilience recovery and commitment growth reinforced each other over the 12-month observation window. We advanced PsyCap theory by demonstrating that resilience’s malleability carries consequences beyond the construct itself, shaping the developmental slope of a motivational attitude in a distinct psychological domain. Third, this study offered methodological proof of concept: parallel-process LGM can answer questions about co-development that neither cross-lagged panels nor simple regression approaches are equipped to address.

For practitioners, the results point toward phase-sensitive intervention rather than a uniform prescription. Resilience support—coaching, workload redistribution, peer networks—should be concentrated during peak implementation strain, when resource drain is steepest and Carnall’s coping-cycle stages of defense and discarding (Carnall, 2007) are most likely to be active. Communication efforts aimed at building affective commitment should begin early and maintain consistency; the linear growth pattern suggests that each wave of credible information compounds upon the last. The differential responsiveness of affective versus continuance commitment implies that organizations gain more from cultivating genuine enthusiasm than from amplifying switching costs. Moreover, that only the affective route plausibly underwrites the sustainability of change once formal implementation ends.

Several limitations temper the conclusions. Our sample was drawn exclusively from Chinese manufacturing firms undergoing operational restructuring; whether the same trajectory shapes and cross-domain couplings hold in service-sector organizations, in Western cultural contexts, or during different types of change (e.g., mergers, leadership transitions) remains an open question. The 6-month interval between waves, while practical, may have been too coarse to capture rapid fluctuations in resilience during the first weeks of implementation—a period when daily stressors peak. All measures relied on self-report, raising the possibility of common method variance inflating within-wave correlations; the sensitivity analysis reported in section 4.1 using an unmeasured latent method construct suggested that our substantive conclusions are robust, but residual bias cannot be ruled out. Related, and more consequential, we emphasize that three-wave LGM supports predictive claims about temporal precedence and slope co-movement but does not, on its own, warrant efficient-cause claims about resilience “driving” commitment—future randomized or quasi-experimental designs will be needed to license that stronger reading (Podsakoff et al., 2024). Finally, three waves suffice for linear and simple quadratic models but cannot support more complex functional forms or formal tests of mediation within the growth curve framework.

Future work should pursue at least three directions. Cross-cultural replications would clarify the boundary conditions of the co-developmental pattern we documented. Experience sampling methods with daily or weekly measurement bursts nested within the broader longitudinal design could capture the micro-dynamics of resilience fluctuation that six-month intervals inevitably smooth over. Most ambitiously, randomized intervention studies—comparing organizations that receive targeted resilience training and communication protocols against control sites—would move the field from correlational growth modeling toward causal inference about how to shape these trajectories deliberately.

## Data Availability

The datasets generated and analysed for this study are available from the corresponding author on reasonable request.
